# Rethinking gadolinium hypersensitivity management: from corticosteroid premedication to evidence-informed alternative GBCA selection

**DOI:** 10.3389/fradi.2026.1894050

**Published:** 2026-10-21

**Authors:** Yuan Yao, Feng Gao

**Affiliations:** Department of Diagnostic Radiology, National Cancer Center/National Clinical Research Center for Cancer/Cancer Hospital, Chinese Academy of Medical Sciences and Peking Union Medical College, Beijing, China

**Keywords:** alternative GBCA selection, anaphylaxis, basophil activation test (BAT), corticosteroid premedication, gadolinium-based contrast agents (GBCAs), hypersensitivity reactions (HSRs), nursing interventions, radiology nursing

## Abstract

Recurrent hypersensitivity reactions (HSRs) to gadolinium-based contrast agents (GBCAs) are increasingly recognized as contrast-enhanced MRI use expands. Current guidelines no longer uniformly recommend routine corticosteroid premedication for prior GBCA reactors, and evidence questions premedication given without agent change: breakthrough reactions occur in a pooled 39% of premedicated patients, and antihistamines can plausibly mask early anaphylaxis warning signs, though direct evidence that premedication *per se* worsens outcomes is lacking. Changing to an alternative GBCA—preferably selected with allergological evaluation when clinically indicated, rather than solely by structural class—is associated with substantially lower recurrence in observational cohorts and one prospective single-arm trial; premedication remains an evidence-limited adjunct in selected circumstances. Skin testing and the basophil activation test (BAT) are specialist adjuncts for identifying culprit and tolerated alternative agents; for moderate-to-severe immediate reactions, current ESUR guidance additionally recommends acute serum tryptase measurement and allergy referral. Delayed reactions, including severe cutaneous adverse reactions (SCAR), are addressed only where they require distinct management: non-SCAR delayed reactions undergo delayed-reading and patch testing (with BAT not applicable and drug provocation testing reserved for selected patients at specialist allergy centres), whereas lifelong avoidance of all GBCAs is reserved for confirmed SCAR, in which drug provocation testing is contraindicated—guideline-derived recommendations outside our formally screened immediate-HSR evidence base. We propose a practical, severity-stratified algorithm for radiology and nursing teams, labeling its elements as evidence-supported, guideline-derived, or author-proposed.

## 1 Introduction

With roughly 50 million contrast-enhanced MRI procedures performed worldwide yearly, even the low baseline HSR rate yields a growing absolute burden: immediate allergic-like events occur in about 9 per 10,000 administrations, with severe reactions near 0.5 per 10,000 (1, 2). Most immediate reactions are non-IgE-mediated, but IgE-mediated mechanisms are increasingly documented through skin tests and basophil activation tests (1, 5). Re-exposure after an initial reaction carries markedly higher risk.

Contrary to a common misconception, the ACR Manual on Contrast Media, the ESUR Contrast Media Safety Committee guidelines (Parts 1 and 2, 2025), and the 2025 ACR–AAAAI consensus statement advocate risk-adapted management and do not endorse universal premedication; the ACR Manual states that premedication prophylaxis is of unproven efficacy (6–8, 30, 31). Breakthrough reactions occur in a pooled 39% (95% CI, 30%–49%) of premedicated patients (10), and antihistamines can plausibly suppress early cutaneous warning signs—recent allergological reviews note this may mask early anaphylaxis without protecting against more serious outcomes (34)—although direct evidence that premedication *per se* delays recognition or worsens outcomes is lacking. Institutional surveys describe variability in MRI-suite resuscitation readiness (11)—a caution where nursing readiness is uneven.

A better-supported alternative is to stop re-administering the culprit agent and use a different GBCA, ideally selected with allergological input (30, 31). Macrocyclic and linear chelates differ in geometry, protein binding, and hapten presentation, which may limit—but does not abolish—cross-reactivity: cross-reactivity has been documented in a substantial proportion of evaluated allergic patients, including macrocyclic–linear combinations, and cannot be reliably predicted from chemical structure alone (13, 16, 28, 30, 32). Prospective and propensity-matched data consistently associate changing the culprit agent with lower recurrence (13–15). This mini-review synthesizes that evidence and offers a practical algorithm—labeling its elements evidence-supported, guideline-derived, or author-proposed—for radiology and nursing teams. The primary focus is immediate HSRs, defined throughout as reactions within 1 h of administration per current ESUR and ACR terminology (8, 30); where cited studies applied wider windows (up to 6 h), this is noted where relevant to comparability. Delayed T-cell-mediated reactions are considered only where they demand distinct management.

## 2 Literature search strategy

This mini-review followed a structured narrative approach. Searches of PubMed and ScienceDirect were last run on September 10, 2026. The PubMed strategy used the following free-text terms: (“gadolinium-based contrast agents” OR GBCA OR gadolinium) AND (“hypersensitivity” OR anaphylaxis OR “skin testing” OR “basophil activation test” OR premedication OR “alternative agent” OR “cross-reactivity”). An equivalent keyword strategy was adapted for the other database: ScienceDirect—gadolinium AND (hypersensitivity OR anaphylaxis) AND (premedication OR “skin test” OR “basophil activation” OR “cross-reactivity” OR “alternative agent”). Google Scholar was not used as a structured bibliographic source; it served as an *ad hoc* tool within the targeted supplementary searches to locate specific records and access full texts. The search spanned 2006–2026. The searches identified 1037 records (PubMed 271, ScienceDirect 766); after duplicate removal in the reference manager (987 unique records) and a title-level relevance scan, supplemented by reference screening of key guidelines and reviews and by targeted searches for specific topics, this yielded the 36 sources cited in this review (original studies, narrative and systematic reviews or meta-analyses, and guidelines or consensus statements). English-language studies reporting GBCA-related immediate HSRs, diagnostic testing, premedication, or alternative agent selection were included; animal-only studies and non-peer-reviewed commentaries were excluded. Delayed T-cell-mediated reactions were not a primary focus of screening; relevant delayed-reaction and SCAR reports, whether retrieved incidentally by these searches or secured through reference screening and targeted searches, are discussed only where distinct management applies (9, 19, 20, 30, 32). Titles and abstracts were screened by the first author (YY); full-text eligibility was assessed independently by two reviewers (YY, FG), with disagreements resolved by consensus. Global pharmacovigilance databases (VigiBase, FAERS, IQVIA-MIDAS) were not queried directly; published peer-reviewed analyses based on these databases (3) were identified through the bibliographic searches described above, and no unpublished or original data were generated, consistent with our Data Availability Statement. No formal risk-of-bias assessment or certainty-of-evidence grading was undertaken; this limitation is restated in the Discussion and is explicitly linked to the cautious strength of all conclusions below.

## 3 Limitations of corticosteroid premedication

Corticosteroid premedication for prior GBCA reactors was imported from iodinated contrast media—particularly high-osmolar agents with far higher anaphylactoid potential—and was never validated with GBCA-specific immunological data (6, 12, 17). Guidelines accordingly adopt risk-adapted rather than universal approaches (6–8).

GBCAs are, however, immunologically distinct—chelated complexes with their own protein-binding, ionicity, and dissociation characteristics. Walker et al.'s meta-analysis found a pooled breakthrough HSR rate of 39% (95% CI, 30%–49%) despite full premedication, with similar rates across GBCA classes (macrocyclic 36% vs. protein-binding linear-ionic 31%) (10); a failure rate of this magnitude, though derived from observational data, argues against premedication as a standalone prevention strategy (10, 14, 15).

Antihistamines given before the scan can suppress early urticaria and mucosal symptoms—the very signs that alert nursing staff to an evolving reaction. This suppression is biologically plausible and clinically relevant, although the available evidence does not directly establish that antihistamine premedication *per se* delays recognition of anaphylaxis or worsens outcomes (4). This underscores the need for adrenaline readiness, trained responders, and close nursing surveillance.

Whether premedication adds value beyond switching is unclear. Walker et al.'s prospective trial found no measurable benefit of adding corticosteroids and antihistamines to a structurally different GBCA (15), and in the Ryoo cohort, single-dose premedication added no benefit to agent change (7.2% vs. 6.1%, *p* = 0.821) (13). In patients with prior severe anaphylaxis, however, a subgroup finding from a single retrospective propensity-matched cohort—a non-randomized subgroup comparison requiring prospective confirmation—suggests switching plus premedication yields lower recurrence than switching alone (14), supporting premedication as an evidence-limited adjunct rather than an established pathway component (14, 30, 31). These findings argue against reflexive premedication as first-line prevention.

## 4 Immunological considerations and the evidence for selecting an alternative GBCA

GBCA-HSRs are driven by IgE-mediated and non-IgE-mediated mechanisms. In IgE-mediated reactions, hapten-specific IgE triggers mast-cell and basophil degranulation on re-exposure to the same or a structurally similar agent—a pathway supported by positive skin tests and BAT in GBCA reactors (1, 5, 24, 26, 34). Non-IgE pathways include direct mast-cell activation via MAS-related G-protein-coupled receptor X2 (MRGPRX2)—demonstrated experimentally for meglumine gadoterate, implicating the meglumine moiety (35)—and complement activation (18, 34).

IgE-mediated cross-reactivity between structurally related agents is the principal driver of recurrent immediate reactions (1, 5, 24), but it is not uniform across the GBCA class and cannot be reliably predicted from chemical structure alone; the ESUR Contrast Media Safety Committee states this explicitly, noting a higher frequency among macrocyclic GBCAs (30). In a series of 28 patients with proven GBCA allergy (11 single-center, 17 published cases), skin-test cross-reactivity was found in approximately 29% overall (18.2% and 35.3%), was confined to immediate reactions, involved other macrocyclic agents or macrocyclic–linear combinations, and was not observed among linear agents (32). In a single-center series, cross-reactivity rates were reported as 27.7% among macrocyclic agents, 5.5% among linear agents, and 5.5% between the two classes (28). Cross-reactivity risk is therefore principally within the macrocyclic group; macrocyclic–linear cross-reactivity is lower but documented, and appears rare between linear agents (28, 32, 33). Two caveats follow: switching to the “opposite” class is not automatically safe (30), and European restrictions on linear GBCAs (nephrogenic-systemic-fibrosis and deposition concerns) limit the macrocyclic-to-linear option, making allergy-guided selection more important (30, 33). What the evidence supports is changing away from the culprit agent—preferably to a molecule identified as tolerated through allergy evaluation—rather than a fixed class-to-class rule (13–15, 30).

A six-year propensity-score-matched cohort of over 150,000 patients showed that changing the GBCA lowered recurrent acute adverse-reaction risk to 14.3% versus 36.9% with re-exposure to the same or related agent (odds ratio 0.32) (14). Ryoo et al. reported recurrent mild immediate reactions in 6.2% after switching to a GBCA of a different molecular structure class versus 11.8% after switching within the same structural class (*n* = 17) and 25.8% with re-exposure to the culprit agent (13). A prospective single-arm trial of empiric switching in documented prior immediate HSR reported recurrence in only 3.7% (15). Taken together, these predominantly observational studies—diverging in design, definitions, severity, comparators, and patient selection, and therefore not directly comparable—consistently associate changing the culprit GBCA with lower recurrence; ESUR Part 2 summarizes this literature as an approximately 75% lower recurrence rate when a GBCA with a different chelate structure is administered (30). However, no randomized head-to-head comparison exists, so these data support agent change as an evidence-informed strategy rather than establishing the superiority of any specific selection rule (Table 1).

**Table 1:** Comparison of corticosteroid premedication and alternative GBCA selection for prevention of recurrent GBCA hypersensitivity.

Feature	Corticosteroid Premedication	Alternative GBCA selection
Mechanism	Broad anti-inflammatory suppression of mast-cell/basophil activation; borrowed from iodinated contrast protocols and not validated for GBCAs (6, 12)	Avoids re-exposure to the culprit agent; cross-reactivity is variable and cannot be predicted from chemical structure alone, so alternative selection should be allergy-guided when clinically indicated (13, 16, 28, 30, 32)
Evidence base	Extrapolated from iodinated contrast; effectiveness for GBCAs unproven in randomized data (6, 10)	Retrospective propensity-score-matched cohort, *n* > 150,000, mixed severities (14); retrospective single-center cohort, prior mild reactors (13); prospective single-arm trial, prior immediate HSR (15); no randomized head-to-head comparison; designs heterogeneous
Breakthrough reaction rate	Pooled 39% despite full premedication (10)	14.3% vs. 36.9% (retrospective, propensity-score-matched, mixed severities) (14); 6.2% vs. 11.8% vs. 25.8% (different vs. same structure vs. culprit re-exposure; retrospective, prior mild reactors) (13); 3.7% (prospective single-arm, prior immediate HSR) (15) — estimates derive from heterogeneous designs and are not directly comparable
Additive benefit of premedication	Baseline strategy	No significant further reduction when added to switching (13, 15); an evidence-limited adjunct in selected circumstances, e.g., prior severe reactors before allergy evaluation is completed (subgroup finding from a single retrospective cohort) (14, 30)
Side effects/drawbacks	Hyperglycemia, fluid retention, scheduling delays; antihistamines may plausibly suppress early warning signs, although direct evidence of delayed recognition is lacking (4, 11)	Minimal; rare non-IgE-mediated or idiosyncratic reactions may still occur (4, 30)
Workflow impact	Requires advance scheduling, patient compliance, additional pharmacy and nursing coordination	Same-day implementation; no pre-visit preparation needed
Current guideline status	Risk-adapted, not universally recommended; efficacy questioned (6–8, 30, 31)	ESUR Part 2 favors changing the culprit agent and allergy-guided alternative selection, particularly after moderate-to-severe reactions (30); the current ACR Manual takes a comparable risk-adapted position (7)
Best suited for	Not recommended as universal first-line; possible targeted use in prior severe reactors under monitoring (14)	Mild prior immediate reactors as first-line prevention; moderate-to-severe reactors with allergy referral and guided selection

Recurrence estimates derive from studies differing in design, populations, severity definitions, and comparators, and are therefore not directly comparable. No randomized severity-stratified head-to-head trial of premedication versus alternative GBCA selection has been published.

## 5 Specialist allergy evaluation: skin testing, BAT, and drug provocation testing

Changing to an alternative GBCA is associated with low recurrence for most mild-to-moderate prior reactors; but when the history is severe, switching has failed, or multiple agents have reacted, diagnostic precision becomes essential. Skin testing and BAT are the two main specialist tools for identifying culprit and tolerated alternative agents; skin testing should be panel-based, including the culprit and candidate alternatives, ideally within 2–6 months of the reaction, when sensitivity is highest (30, 34). Immediate-reading tests are assessed at 15–20 min, delayed readings and patch tests at 24–48 h and beyond (19, 21).

Skin testing follows the EAACI/ENDA protocol: prick testing with undiluted contrast first, followed by intradermal testing at increasing dilutions if prick tests are negative (21). Positivity rates range from 15% to 58%, higher in more severe phenotypes (24, 28). Low-dose provocation tests using one-tenth of the usual injected dose carry a negative predictive value of approximately 86%, and newer rapid challenge protocols that approximate clinical bolus administration reach 89% (22, 23, 33); rapid, high-flow protocols may better capture non-IgE, MRGPRX2-mediated mechanisms (30, 34). Drug provocation testing (DPT) in controlled specialist settings remains the reference standard; guidelines advise DPT when safe alternatives cannot be identified by skin testing or BAT, when the culprit agent tests negative, and when tolerance of a candidate alternative requires confirmation, and only skin-test-negative agents should be challenged (1, 30, 34).

BAT is a specialist adjunct available only at specialized drug-allergy centers and is reserved for selected patients—particularly those with moderate-to-severe reactions or in whom skin testing and challenge are contraindicated (30). It measures CD63 and CD203c upregulation on basophils after *ex vivo* GBCA exposure (24, 25). For contrast media generally, ESUR Part 2 reports specificity above 90% but sensitivity typically only slightly above 50%, partly because basophils express little MRGPRX2 (30); GBCA-specific single-center estimates are higher (sensitivity approximately 68%, specificity 93%, from small single-center series) but should not be extrapolated as general diagnostic performance (24, 34). A positive result is informative, whereas a negative result does not exclude sensitization. Standardization is ongoing—an EAACI task force has published a consensus protocol and position paper (27, 36)—but inter-laboratory variability persists. Combining BAT with skin testing may increase diagnostic yield in complex cases; however, BAT positivity reflects *ex vivo* basophil activation and does not by itself establish a specific non-IgE mechanism (26, 27).

## 6 Proposed clinical management algorithm

Synthesizing the evidence and current guidelines (7, 30), we propose a severity-stratified, phenotype-based algorithm (Figure 1) that distinguishes immediate from delayed pathways and, within the delayed pathway, non-SCAR delayed hypersensitivity from confirmed GBCA-associated SCAR; each element is labeled as evidence-supported (ES), guideline-derived (GD), or author-proposed (AP). Immediate and delayed HSRs must not be managed together (9, 19, 20). Within the immediate pathway, mild and moderate-to-severe reactions are managed as parallel severity branches rather than sequential steps, and a reaction recurring after an initially mild episode moves the patient to the moderate-to-severe branch (Figure 1).

**Figure 1:**
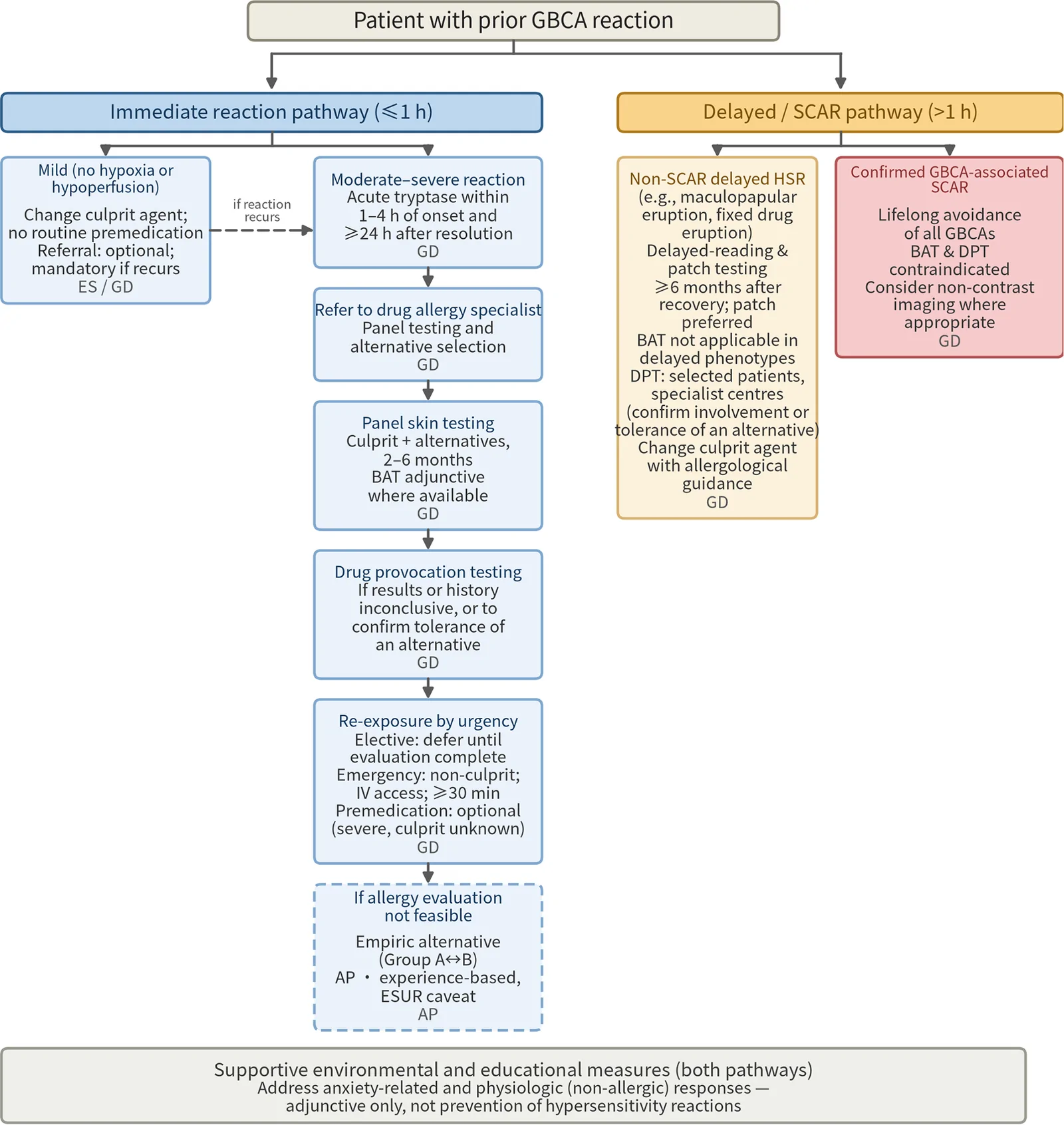
Proposed clinical algorithm for managing patients with prior gadolinium-based contrast agent (GBCA) hypersensitivity, structured after current guidelines (30). Pathways are first separated by phenotype: immediate reactions are defined as those occurring within 1 h of administration, and delayed reactions follow a separate specialist pathway in which non-SCAR delayed hypersensitivity is distinguished from confirmed GBCA-associated SCAR. Within the immediate pathway, mild and moderate-to-severe reactions are managed as parallel severity branches rather than sequential steps: mild reactions (no hypoxia or hypoperfusion) are managed by changing the culprit agent to an alternative GBCA without routine premedication, with optional allergy referral where specialist capacity allows and mandatory referral if any reaction recurs (ES/GD); moderate-to-severe reactions trigger acute serum tryptase measurement (within 1–4 h of symptom onset and ≥24 h after resolution), referral to a drug allergy specialist, panel-based skin testing including the culprit and candidate alternatives (ideally within 2–6 months), adjunctive BAT where available, and drug provocation testing when results or history remain inconclusive or when tolerance of a candidate alternative requires confirmation (GD). A reaction recurring after an initially mild episode moves the patient to the moderate-to-severe branch (dashed arrow). Elective imaging is postponed for allergy evaluation; emergency re-exposure uses a non-culprit agent with full backup and at least 30 min of nursing observation with intravenous access in place, with premedication as a guideline-optional measure only for severe reactions to an unknown culprit. Where allergy evaluation is not feasible, an empiric alternative may be chosen (e.g., GBCA Group A↔B; dashed box), explicitly labeled as an author-proposed (AP), experience-based option, with the caveat that no evidence-supported empiric-switching recommendation exists (30). Within the delayed pathway, non-SCAR delayed hypersensitivity (e.g., maculopapular eruption, fixed drug eruption) is evaluated with delayed-reading and patch testing (≥6 months after recovery; patch preferred); BAT is not applicable in delayed phenotypes, whereas drug provocation testing may be considered in selected patients at specialist allergy centres to confirm involvement of a contrast medium or tolerance of an alternative when appropriate; the branch is managed by changing the culprit agent with allergological guidance; lifelong avoidance of all GBCAs is reserved for confirmed GBCA-associated SCAR, in which BAT and drug provocation testing are contraindicated, with non-contrast imaging considered where appropriate (GD). Supportive environmental and educational measures address anxiety-related, non-allergic responses and are adjunctive only. ES, evidence-supported; GD, guideline-derived; AP, author-proposed; SCAR, severe cutaneous adverse reaction; BAT, basophil activation test; DPT, drug provocation test; IV, intravenous.

For mild immediate reactions without hypoxia or hypoperfusion (limited urticaria, localized pruritus, mild bronchospasm, transient nausea), the recommendation—consistent with guideline direction and observational data (ES/GD)—is to change the culprit agent to an alternative GBCA without routine premedication; where feasible, the alternative is selected with allergological input (13–15, 30). Patients with moderate reactions, recurrent reactions, or an unclear culprit history should additionally be offered allergy specialist evaluation (GD). Standard emergency preparedness, including nursing readiness for anaphylaxis, remains in place.

For moderate-to-severe immediate reactions—anaphylaxis requiring adrenaline, angioedema with airway compromise, hypotensive syncope, severe bronchospasm with hypoxemia—the pathway follows current guidance (GD) (7, 30): (i) measure serum tryptase acutely, within 1–4 h of symptom onset and ≥24 h after resolution, to confirm mast-cell activation; (ii) refer to a drug allergy specialist; (iii) in the specialist setting, perform panel-based skin testing (culprit and candidate alternatives), BAT adjunctively where available, and drug provocation testing when results or history are inconclusive or tolerance confirmation is needed; and (iv) manage re-exposure by urgency: elective imaging waits until allergy evaluation is complete; emergency imaging uses a non-culprit agent under full emergency backup, with trained responders present and at least 30 min of nursing observation with intravenous access (30). Premedication is guideline-optional only for emergency re-exposure after a severe reaction to an unidentified culprit; otherwise it remains an evidence-limited adjunct rather than an established component of the pathway (14, 30, 31). Any patient with documented GBCA-associated SCAR should avoid all GBCAs indefinitely—a guideline-derived recommendation (8, 9, 30) that lies outside our formally screened immediate-HSR evidence base. Reported GBCA-associated SCARs to date comprise isolated DRESS and acute generalized exanthematous pustulosis cases, most involving gadobutrol (34); diagnosis rests on clinical phenotype and specialist delayed-reading and patch testing (patch testing preferred, ≥6 months after full recovery); while BAT and immediate-reading tests are not applicable, and drug provocation testing is contraindicated (9, 30, 34).

Within the delayed pathway, non-SCAR delayed hypersensitivity—typically maculopapular eruptions or fixed drug eruptions—is shown as a branch separate from confirmed SCAR (Figure 1): it is evaluated with delayed-reading and patch testing (≥6 months after full recovery; patch preferred); BAT is not applicable in these delayed phenotypes, whereas drug provocation testing may be considered in selected patients at specialist allergy centres to confirm involvement of a contrast medium or tolerance of an alternative when appropriate; and it is managed by changing the culprit agent with allergological guidance rather than lifelong avoidance of all GBCAs, which is reserved for confirmed SCAR, in which BAT and drug provocation testing are contraindicated (9, 30).

When timely allergy evaluation is not feasible, an empiric alternative may be required. ESUR Part 2 offers suggestions based on practical experience, using a chelating-agent classification in which all currently recommended GBCAs are macrocyclic (Group A, gadoterate meglumine; Group B, gadoteridol and gadobutrol; Group C, gadopiclenol): for a culprit agent from Group A, choose one from Group B, and vice versa (empiric advice for Group C being insufficient); when the culprit is unknown, use an agent different from the one routinely administered. The historically used linear agents—gadopentetate dimeglumine, gadodiamide, and gadoversetamide—are not part of these empiric suggestions. The guideline explicitly cautions that it cannot provide evidence-supported recommendations for switching based on practical experience alone; allergy-guided selection remains superior whenever feasible (30, 34). If testing shows reactivity to multiple agents, or severe reactions have involved agents from different groups, non-contrast MRI or alternative imaging (contrast-enhanced CT if not contraindicated, ultrasound, nuclear medicine) is preferred—though a well-indicated enhanced examination should not be denied if no alternative exists (30).

## 7 Discussion

Routine premedication fails in a meaningful minority, adds no clear benefit when switching is already planned, and can obscure early anaphylaxis signs where nursing and emergency readiness are uneven (4, 10, 11, 15). Changing the culprit GBCA is mechanistically plausible and consistently associated with lower recurrence in observational studies (13–16, 30), although cross-reactivity patterns cannot be predicted from structural class alone (28, 30, 32, 33). These observations do not preclude a targeted role for premedication (14).

Current guidelines converge on risk-adapted management but differ in scope and formality. ESUR Part 2 (30)—the guideline most directly applicable to GBCAs—does not endorse routine premedication (optional only in emergencies after a severe reaction to an unidentified culprit), recommends acute tryptase measurement within 1–4 h of moderate-to-severe immediate reactions with a baseline sample at least 24 h after resolution, referral of at least all moderate-to-severe reactions to a drug allergy specialist, panel-based skin testing ideally within 2–6 months, drug provocation testing when skin testing or BAT cannot identify a safe alternative, and allergy-guided selection of alternatives; it explicitly cautions that cross-reactivity cannot be predicted from chemical structure alone and that it cannot make evidence-supported recommendations for switching based on practical experience alone. The current ACR Manual (7) likewise does not advocate universal premedication for prior contrast reactors. The 2025 ACR–AAAAI consensus (31)—developed for iodinated contrast media rather than GBCAs—corroborates the field-wide de-emphasis of premedication (none after mild index reactions, switching preferred when feasible; hospital-based premedication reserved for severe reactors without alternative imaging) and specifically advises against premedication for iodinated contrast on the basis of an isolated prior GBCA reaction; its direct bearing on GBCA management is therefore limited, and ESUR Part 2 and the ACR Manual remain the primary GBCA-relevant sources. Our algorithm aligns with both on the non-universality of premedication and the central role of changing the culprit agent; the severity-stratified triage, referral thresholds, and empiric fallback in Figure 1 are our operational extensions (AP) for settings without immediate allergy referral.

Several gaps remain: randomized comparisons of premedication versus alternative selection in severity-stratified cohorts are lacking (13–15); BAT standardization remains incomplete (27); non-IgE mechanisms are incompletely mapped; post-switching follow-up is thin.

Delayed hypersensitivity merits separate consideration: maculopapular rashes, fixed drug eruptions, and SCAR are typically T-cell-mediated and may not follow the same structural-class rules (1, 9, 20). Whether premedication has any role is unresolved; current guidance treats GBCA-associated SCAR as an indication for lifelong avoidance of all GBCAs (9, 30)—a guideline-derived recommendation outside our formally screened immediate-HSR evidence base.

Limitations of this review include its structured-narrative design, the absence of formal risk-of-bias assessment or certainty-of-evidence grading, and a predominantly observational, heterogeneous evidence base; confidence in all recommendations therefore remains limited, and they should be interpreted accordingly.

Beyond pharmacologic prevention, supportive environmental and educational measures—such as patient-friendly audiovisual systems, which reduced early post-injection adverse reactions from 1.6% to 0.7% (29)—address anxiety-related, non-allergic responses; they complement, but must not be conflated with, HSR prevention.

## 8 Conclusion

The evidence reviewed here indicates that routine corticosteroid premedication for prior GBCA reactors—a practice inherited largely from another contrast class—is not validated for GBCAs and is not universally recommended by current ACR and ESUR guidance. Changing away from the culprit agent—selected with allergological guidance for clinically significant reactions, since cross-reactivity cannot be predicted from structural class alone—is consistently associated with lower recurrence across observational and single-arm prospective data; absent randomized, severity-stratified trials, confidence in the evidence remains limited because the available evidence is predominantly observational and heterogeneous. Premedication may retain an evidence-limited adjunctive role in selected circumstances, such as unavoidable re-exposure in prior severe reactors before allergy evaluation is complete. Radiology departments should adopt structured alternative-agent selection as first-line prevention for mild reactions, measure acute serum tryptase and refer for allergy evaluation after moderate-to-severe reactions, reserve panel-based skin testing, adjunctive BAT, and drug provocation testing for specialist pathways, and invest in nursing measures and emergency preparedness as complementary safeguards. Delayed reactions—addressed here on the basis of guideline recommendations rather than the formally screened immediate-HSR evidence—require specialist evaluation with delayed-reading and patch testing, whereas lifelong avoidance of all GBCAs is reserved for confirmed GBCA-associated SCAR.

## Data Availability

The datasets analyzed for this study are available in the published articles cited in the reference list.
